# Association of workplace stressors prior to infection and the development of Long COVID among workers during the COVID-19 pandemic: a cohort study in Japan

**DOI:** 10.1093/joccuh/uiae062

**Published:** 2024-11-28

**Authors:** Yu Igarashi, Seiichiro Tateishi, Arisa Harada, Ayako Hino, Mayumi Tsuji, Hajime Ando, Shinya Matsuda, Yoshihisa Fujino, Koji Mori

**Affiliations:** Disaster Occupational Health Center, Institute of Industrial Ecological Sciences, University of Occupational and Environmental Health, 1-1 Iseigaoka Yahatanishi-ku Kitakyushu, Fukuoka 807-8555, Japan; Disaster Occupational Health Center, Institute of Industrial Ecological Sciences, University of Occupational and Environmental Health, 1-1 Iseigaoka Yahatanishi-ku Kitakyushu, Fukuoka 807-8555, Japan; Department of Occupational Medicine, School of Medicine, University of Occupational and Environmental Health, 1-1 Iseigaoka Yahatanishi-ku Kitakyushu, Fukuoka 807-8555, Japan; Department of Mental Health, Institute of Industrial Ecological Sciences, University of Occupational and Environmental Health, 1-1 Iseigaoka Yahatanishi-ku Kitakyushu, Fukuoka 807-8555, Japan; Department of Environmental Health, School of Medicine, University of Occupational and Environmental Health, 1-1 Iseigaoka Yahatanishi-ku Kitakyushu, Fukuoka 807-8555, Japan; Department of Work Systems and Health, Institute of Industrial Ecological Sciences, University of Occupational and Environmental Health, 1-1 Iseigaoka Yahatanishi-ku Kitakyushu, Fukuoka 807-8555, Japan; Department of Preventive Medicine and Community Health, School of Medicine, University of Occupational and Environmental Health, 1-1 Iseigaoka Yahatanishi-ku Kitakyushu, Fukuoka 807-8555, Japan; Department of Environmental Epidemiology, Institute of Industrial Ecological Sciences, University of Occupational and Environmental Health, 1-1 Iseigaoka Yahatanishi-ku Kitakyushu, Fukuoka 807-8555, Japan; Department of Occupational Health Practice and Management, Institute of Industrial Ecological Sciences, University of Occupational and Environmental Health, 1-1 Iseigaoka Yahatanishi-ku Kitakyushu, Fukuoka 807-8555, Japan

**Keywords:** COVID-19, Japan, Long COVID, job content questionnaire, workers, workplace stressor

## Abstract

**Objectives:**

Long COVID is a global health concern. Although various risk factors are known, the link between workplace factors and Long COVID is underexplored. We conducted a cohort study to examine the impact of preinfection workplace stressors on Long COVID among Japanese workers, with the aim of improving understanding of its disease process and inform workplace management.

**Methods:**

This prospective cohort study used online survey data collected in December 2020 and December 2022 from 1539 participants with no initial infection history but later reported COVID-19. Workplace stressors were assessed at baseline using the Job Content Questionnaire, which measured job demands, job control, and social support. At the follow-up survey, COVID-19 infection was determined by self-reported positive SARS-CoV-2 test by polymerase chain reaction or antigen, and development of Long COVID was defined as symptoms persisting for over 2 months. Univariate and multivariate logistic regression analyses estimated odds ratios (ORs) for the association between workplace stressors and the development of Long COVID.

**Results:**

Of the participants, 248 reported Long COVID. Univariate analysis showed that both high job demands (OR: 1.55; 95% CI, 1.09-2.20) and poor job control (OR: 1.50; 95% CI, 1.04-2.18) increased the odds. Poor supervisor support (OR: 1.73; 95% CI, 1.16-2.58) and co-worker support (OR: 1.59; 95% CI, 1.13-2.23) were also significant. On multivariate analysis, job demands remained significantly associated with Long COVID.

**Conclusions:**

Our findings indicate that preinfection workplace stressors may contribute to Long COVID development in workers. Managing workplace stressors effectively could be a preventive measure.

## Key points

Since the disease process of Long COVID, which follows COVID-19 infection, is not well understood, and research on the role of workplace environments is limited, our study aimed to investigate the relationship between preinfection workplace stressors and the development of Long COVID symptoms among Japanese workers.

Our findings indicate significant correlations between elevated job demands and poor job control, supervisor support, and co-worker support and the development of Long COVID. Our findings suggest that reducing workplace stressors might help mitigate the risk of prolonged symptoms.

Additionally, our analysis showed that the inclusion of the K6 scale, which measures psychological distress, reduced the statistical impact of job control and social support. This suggests that mental health issues, exacerbated by workplace stressors, could be a contributing factor to Long COVID development. Therefore, addressing mental health and providing strong social support in the workplace could be crucial in preventing Long COVID. These results underscore the importance of implementing workplace stressor mitigation strategies and maintaining effective workplace infection control measures during future COVID-19 and similar pandemics to protect worker health and well-being.

## 1. Introduction

It has been reported that 10%-35% of people with COVID-19 develop symptoms after infection.^1^^-^^4^ The World Health Organization (WHO) defines post-COVID-19 syndrome as the continuation or onset of new symptoms 3 months after initial infection and the persistence of these symptoms for at least 2 months.^5^ The National Institute for Health and Clinical Excellence (NICE) defines it as a condition that persists for 12 weeks after initial infection,^6^ whereas the Centers for Disease Control and Prevention (CDC) defines it as a post-COVID condition with symptoms that persist for at least 3 months after infection and are not caused by other diseases.^7^ The Japanese Ministry of Health, Labour, and Welfare defines post-COVID-19 syndrome as a condition that lasts at least 2 months and is not caused by other diseases.^8^ Here, we use the term “Long COVID.”

It is estimated that 650 million people are currently infected with the new coronavirus and that 10% of these, or 65 million, are suffering from persistent symptoms, making it a serious public health problem.^9^ The CDC estimates that 100 million people in the working-age population have been infected and 31 million people have Long COVID.^7^ Long COVID has been reported to be associated with poor performance, workload adjustments, missed workdays, and retirement, resulting in significant losses to the global workforce.^10^^-^^18^

To date, many studies have reported a variety of risk factors for Long COVID.^19^^-^^24^ Some studies have reported consistent results, whereas those of others have been widely varying. A 2022 umbrella review cites female gender, comorbidities, severity of acute illness, and obesity as risk factors.^25^ Some also report that mental illness is a risk factor for Long COVID, as reported in a UK adult population^26^ and among a population of nurses.^27^ To our knowledge, however, no study has investigated the association of workplace stressors with Long COVID. Given the significant impact of Long COVID on the working-age population, it is necessary to examine the association of the work environment of workers and the development of Long COVID.

Here, to better understand the disease process of Long COVID and strategies to alleviate symptoms, we conducted a cohort study of workers during the pandemic, with particular focus on the association between preinfection workplace stressors and the development of Long COVID in the Japanese workforce.

## 2. Methods

### 2.1. Study design and participants

This study was a prospective cohort study using data from the Collaborative Online Research on the Novel-coronavirus and Work (CORoNaWork) Project.^28^ The baseline survey was conducted in December 2020 and the follow-up survey in December 2022; the subjects were monitors of an internet research company (Cross Marketing Inc) who agreed to participate and met the conditions for participation. To examine the association between the development of Long COVID in infected individuals and workplace stressors, we defined the target population as those with a history of COVID-19 infection, specifically those who were not infected at baseline but became infected during follow-up.

The baseline survey was conducted in December 2020. Of the approximately 4.7 million preregistered monitors, invitations to participate in the study were sent to 600 000 individuals with a history of active participation. Of these, about 55 000 met the participation requirements. Survey participants were individuals between the ages of 20 and 60 employed at the time of the survey. Stratified sampling was conducted to ensure equal distribution by gender, occupation (office workers and non–office workers), and region of residence in order to balance the survey population. Forty-seven prefectures of residence were included and divided into 5 levels according to COVID-19 infection rate. A total of 33 302 individuals participated in the study. After excluding those who gave fraudulent responses, 27 036 participants were enrolled. The criteria for fraudulent responses were as follows: response time of less than 6 minutes, weight of less than 30 kg, height of less than 140 cm, multiple contradictory responses regarding family members living in the same household, and a fraudulent response to a question requiring the selection of the third-highest number of 4 numbers presented. After excluding 198 participants with a history of COVID-19 infection at baseline, the study population was reduced to 26 841 participants.

The follow-up survey was conducted in December 2022, with 13 079 (48.7%) of the 26 841 participants responding. Respondents who had tested positive by polymerase chain reaction (PCR) or antigen test for COVID-19 were considered for analysis, resulting in a final sample size of 1539.

The baseline survey asked participants questions about sex, age, marital status, education, income, smoking and drinking habits, exercise habits (of at least 30 minutes), sleep quality, breakfast frequency, medical history, psychological status, and workplace stressors. Medical history included diabetes mellitus, hypertension, depression, asthma, otolaryngological disorders, and dermatosis currently being treated. For drinking habits, participants were asked how often they consumed alcohol per week. The question regarding exercise habits asked about the frequency of days when participants engaged in light, sweaty exercise for 30 minutes or more per day. Sleep quality was assessed with the question, “Do you get enough sleep?” Psychological status was assessed using the K6 scale.^29^^,^^30^ A cutoff point of 10 was adopted to screen for mood and anxiety disorders.^31^ Workplace stressors were assessed by the Job Content Questionnaire (JCQ), which measured job stressors and social support.

In the follow-up survey, we investigated the history of COVID-19 infection, frequency of vaccination, presence or absence of Long COVID, and symptoms of Long COVID.

This study was approved by the Ethics Committee of the University of Occupational and Environmental Health Sciences (ref. nos R2-079 and R3-006). The Strengthening the Reporting of Observational Studies in Epidemiology (STROBE) guideline for cross-sectional studies was used to prepare this report.^32^

### 2.2. Assessment of workplace stressors at baseline survey

We assessed workplace stressors, including job stressors and social support, using the JCQ at baseline survey. The JCQ was developed by Karasek et al^33^ and is based on the job demands-control (or demands-control-support) model. The reliability and validity of the Japanese version of the JCQ have been demonstrated by Kawakami et al.^34^ We used the 5-item job demands scale (score range 12-48), 9-item job control scale (score range 24-96), 4-item supervisor support scale (score range 4-12), and 4-item co-worker support scale (score range 4-12). Each item was rated on a 4-point scale (1 = strongly disagree, 4 = strongly agree). Cronbach a in this sample was .62, .75, .95, and .91, respectively. For comparison using logistic regression analysis we divided job stressors and social support into 3 groups—low, middle, and high—based on the first and third quartiles. The job demands and job control scores were dichotomized by the median and then divided into high-strain (high demand/low control), passive (low demand/low control), active (high demand/high control), and low-strain (low demand/high control). In accordance with findings that the risk of depression is lowest in the low-strain group, followed by the active, passive, and high-strain groups, the job strain groups were created in that order.

### 2.3. Assessment of COVID-19 infection and Long COVID at follow-up survey

A history of COVID-19 infection was determined by a response of indicating a positive result on a PCR or antigen test. Long COVID was defined as a “yes” response to the question, “Have you had any symptoms lasting more than 2 months after infection with COVID-19?” using the WHO definition as a reference.^5^ The assessment of Long COVID was conducted using nonvalidated question items, as a standardized questionnaire for Long COVID has not been established. The development of these items was guided by the expertise of co-author S.T., who is one of the authors of the guidelines for managing post-COVID conditions in Japan.^8^

### 2.4. Statistical analysis

In the analyses, workplace stressors, including job stressors, and social support were treated as the exposure variables, whereas the development of Long COVID was treated as the outcome variable. Using a multilevel logistic regression model, with individual respondents nested within prefectures, univariate and multivariate odds ratios (ORs) were estimated (Model 1), with adjustment for age, sex, body mass index (BMI), household income, educational status, marital status, COVID-19 vaccination status, and past medical history (Model 2). Additionally, the ORs were further adjusted for the K6 scale in Model 3. Inclusion of the K6 scale had a notable impact on the results, prompting the development of a separate model adjusted specifically for this variable. A *P* value of less than .05 was considered statistically significant. All analyses were performed using R version 4.2.2 (R Foundation for Statistical Computing, Vienna, Austria; https://www.r-project.org/).

## 3. Results

The participants in this study are characterized in Table 1. Of the 1539 participants analyzed, 248 reported having Long COVID.

**Table 1:** Characteristics of the participants at the baseline survey.

**Characteristic**	**Infected workers without Long COVID (*n* = 1298)**		**Infected workers with Long COVID (*n* = 248)**		***P* value**
**Age, mean (SD), y**	47.6 (9.9)		47.9 (9.2)		.654
**Sex (men), *n* (%)**	796 (61.7)		138 (55.6)		.088
**Marital status (married), *n* (%)**	885 (68.6)		169 (68.1)		.761
**Education status, *n* (%)**					.030
**Junior high school**	22 (1.7)		2 (0.8)		
**High school**	314 (24.3)		79 (31.9)		
**Vocational school/college, university, graduate school**	955 (74.0)		167 (67.3)		
**Annual equivalent household income (JPY), *n* (%)**					<.001
**£2 990 000**	116 (9.0)		48 (19.4)		
**3 000 000-4 990 000**	276 (21.4)		59 (23.8)		
**5 000 000-6 990 000**	287 (22.2)		48 (19.4)		
**7 000 000-8 990 000**	249 (19.3)		42 (16.9)		
**9 000 000**	363 (28.1)		51 (20.6)		
**Current smoker, *n* (%)**	962 (74.5)		179 (72.2)		.489
**BMI, mean (SD), kg/m** ^**2**^	22.6 (3.5)		22.7 (3.5)		.664
**Alcohol (>4 d/wk), *n* (%)**	860 (66.6)		168 (67.7)		.786
**Sleep (insufficient), *n* (%)**	735 (56.9)		122 (49.2)		.029
**Exercise (>4 d/wk), *n* (%)**	141 (10.9)		22 (8.9)		.396
**Breakfast frequency (>4 d/wk), *n* (%)**	930 (72.0)		170 (68.5)		.299
**Medical history, *n* (%)**					
**Diabetes mellitus**	50 (3.9)		15 (6.0)		.165
**Hypertension**	155 (12.0)		31 (12.5)		.911
**Depression**	60 (4.6)		30 (12.1)		<.001
**Asthma**	26 (2.0)		19 (7.7)		<.001
**Otolaryngological disorders**	48 (3.7)		24 (9.7)		<.001
**Dermatosis**	56 (4.3)		26 (10.5)		<.001
**Psychological status, *n* (%)**					
**K6 (>10)**	209 (16.2)		76 (30.6)		<.001

Table 1 shows the characteristics of participants at baseline and analysis by the presence or absence of Long COVID. Education status, low annual equivalent household income, insufficient sleep, medical history (depression, asthma, otorhinological disorders, and dermatosis), and psychological status were significantly associated with Long COVID.

Table 2 shows the characteristics of the participants at follow-up. There were no statistical differences by vaccination status. The most common symptoms were respiratory symptoms (71.0%) and tiredness, fatigue, and muscle weakness (58.9%).

**Table 2:** Characteristics of the participants at the follow-up survey.

**Characteristic**	**Infected workers without Long COVID** **(*n* = 1298)**		**Infected workers with Long COVID** **(*n* = 248)**	
**Frequency of vaccination, *n* (%)**				
**1**	48 (3.7)		15 (6.0)	
**2**	427 (33.1)		72 (29.0)	
**3**	527 (40.8)		102 (41.1)	
**4**	170 (13.2)		23 (9.3)	
**5**	9 (0.7)		4 (1.6)	
**6**	110 (8.5)		32 (12.9)	
**Symptom of Long COVID, *n* (%)**				
**Fever (37.0°C or higher)**			90 (36.2)	
**Respiratory symptoms**			176 (70.9)	
**Physical pain**			91 (36.6)	
**Cardiovascular symptoms**			77 (31.0)	
**Tiredness, dullness of the body, muscle weakness**			146 (58.8)	
**Abnormal taste or sense of smell**			74 (29.8)	
**Decreased ability to think and concentrate**			79 (31.8)	
**Insomnia, anxiety, mood swings**			70 (28.2)	
**Skin problems (skin rash, itching), hair loss**			59 (23.7)	
**Others**			143 (57.6)	

Table 3 shows the association between job stressors, social support, and the development of Long COVID. In the univariate model (Model 1), job demands had an OR of 1.55 (95% CI, 1.09-2.20; *P* = .015) for high compared with low, job control had an OR of 1.50 (95% CI, 1.04-2.18; *P* = .031), supervisor support had an OR of 1.73 (95% CI, 1.16-2.58; *P* = .006), and co-worker support had an OR of 1.59 (95% CI, 1.13-2.23; *P* = .006) for low (poor) compared with high (good), showing a significant association with Long COVID. In the model adjusted for age, sex, household income, educational status, marital status, BMI, COVID-19 vaccination status, and medical history (diabetes mellitus, hypertension, depression, asthma, otolaryngological disorders, and dermatosis) (Model 2), job demands had an OR of 1.60 (95% CI, 1.11-2.30; *P* = .011) for high compared with low, and supervisor support had an OR of 1.50 (95% CI, 1.00-2.38; *P* = .045) for low (poor) compared with high (good), showing a significant association with Long COVID. In the model further adjusted for K6 (Model 3), job demands was significantly associated with Long COVID with an OR of 1.45 (95% CI, 1.00-2.11; *P* = .048) for high compared with low. For the job strain group, high strain was significantly associated with Long COVID compared with low strain, with an OR of 1.58 (95% CI, 1.07-2.34; *P* = .023) and 1.54 (95% CI, 1.02-2.29; *P* = .045) in Models 1 and 2, respectively.

**Table 3:** Odds ratios (ORs) for univariate and multivariate models of Long COVID development.

	**Univariate Model 1** ^**a**^				**Multivariate Model 2** ^**b**^				**Multivariate Model 3** ^**c**^ **(Model 2 + K6)**			
**Variables**	**OR**	**95% CI**		***P* value**	**OR**	**95% CI**		***P* value**	**OR**	**95% CI**		***P* value**
**Job stressor**												
**Job demands**												
**Low**	ref				ref				ref			
**Middle**	1.45	1.03-2.03		.031	1.58	1.12-2.23		.099	1.53	1.08-2.17		.017
**High**	1.55	1.09-2.20		.015	1.60	1.11-2.30		.012	1.45	1.00-2.11		.048
**Job control**												
**High (good)**	ref				ref				ref			
**Middle**	1.00	0.71-1.40		.998	0.97	0.68-1.37		.850	0.94	0.66-1.33		.723
**Low (poor)**	1.50	1.04-2.18		.031	1.32	0.89-1.95		.161	1.26	0.86-1.73		.248
**Job strain group** ^**d**^												
**Low-strain**	ref				ref				ref			
**Active**	1.11	0.73-1.69		.626	1.08	0.69-1.67		.658	1.05	0.68-1.63		.791
**Passive**	1.11	0.77-1.62		.575	1.07	0.73-1.56		.640	1.08	0.73-1.60		.599
**High-strain**	1.58	1.07-2.34		.023	1.54	1.02-2.29		.045	1.40	0.93-2.12		.135
**Social support**												
**Supervisor support**												
**High (good)**	ref				ref				ref			
**Middle**	1.14	0.45-0.98		.380	1.10	0.81-1.61		.534	1.04	0.75-1.51		.809
**Low (poor)**	1.73	1.16-2.58		.006	1.50	1.00-2.38		.049	1.34	0.89-2.11		.160
**Co-worker support**												
**High (good)**	ref				ref				ref			
**Middle**	0.96	0.68-1.35		.819	0.92	0.65-1.31		.615	0.68	0.63-1.24		.445
**Low (poor)**	1.59	1.13-2.23		.006	1.41	0.99-2.00		.052	0.75	1.29-1.95		.157

a Model 1: adjusted for age, sex.

b Model 2: adjusted for age, sex, body mass index, household income, educational status, marriage status, COVID-19 vaccination, and medical history (diabetes mellitus, hypertension, depression, asthma, otolaryngological disorders, dermatosis).

c Model 3: further adjusted for K6.

d High-strain: high demand/low control; passive: low demand/low control; active: high demand/high control; low-strain: low demand/high control.

## 4. Discussion

This study identified an association between development of Long COVID and preinfection workplace stressors, which include job demands, job control, supervisor support, and co-worker support. Analysis by job strain group also showed an association with Long COVID. To our knowledge, this is the first study to show an association between workplace stressors and the development of Long COVID.

The mechanisms behind this association are unknown, but multiple pathways may be present. Many studies have reported an association between poor mental health prior to infection and Long COVID.^26^^,^^27^ It has been suggested that stress interferes with the immune system, affects hormones and the signaling pathways that regulate the nervous system, and exacerbates postinfection symptoms.^35^ In particular, workplace stressors are an important component of daily life, and persistent stress can have a significant impact on an individual’s mental state.^36^ Workplace stressors may also affect sleep and eating patterns, which may be associated with the development of Long COVID. In our present study, however, the association between workplace stressors and Long COVID remained even after adjustment for variables related to gender, age, BMI, household income, educational status, marital status, vaccination, and medical history, suggesting that workplace stressors may be an independent factor in the development of Long COVID. Our analysis in Model 3 indicates that the statistical significance of the odds of developing Long COVID were partially eliminated after adjustment for mental health factors, suggesting that mental health issues may be a pathway through which workplace stressors contribute to the development of Long COVID.

An important feature of Long COVID is that it also develops at a mature age, when people have jobs and families. The symptoms of Long COVID can accordingly have a significant impact on the social lives of those affected. Many patients have reported that Long COVID interferes with their work and daily lives, causing many workers to lose their job, change jobs, or take a leave of absence.^11^^-^^16^^,^^18^ Our present findings suggest that improving workplace stressors and working in a good mental state during the pandemic may have had a preventive effect on the development of Long COVID.

Reports on risk factors for the development of Long COVID in Japan are limited; in particular, regional and racial factors as causes of Long COVID also remain unknown. Clarifying the risk factors of Long COVID in different countries would likely help elucidate the disease process. In a study limited to the Japanese population, Sugiyama et al^10^ reported that female sex and advanced age were risk factors for Long COVID, whereas Terai et al^37^ reported that female sex, middle age (41-64 years), oxygen requirements, and serious conditions during hospitalization were risk factors. Kataoka et al^38^ reported preexisting psychiatric disorders as a risk factor for Long COVID, and Hazumi et al^39^ reported that patients with psychological distress had higher odds of Long COVID. On comparison of these previous and our present findings, common factors include psychological distress, but not female sex and age. Further studies on risk factors for Long COVID in the Japanese population are needed.

Because Long COVID is associated with significant financial loss, arising, for example, due to poor performance, leave of absence, and retirement, addressing the issue of Long COVID is important from a workforce retention perspective.^10^^-^^18^ From a prevention perspective, implementing strategies to reduce stress in the workplace during future COVID-19 pandemics may contribute to a reduction in the incidence of Long COVID. Taking workplace stressors into account as a new criterion for assessing the risk of Long COVID in specific occupations might also be warranted. Additionally, considering workplace stressors as a risk factor may aid in the development of public health strategies to address Long COVID in high-risk occupations, ultimately promoting better occupational health management. Doing so may also support the development of strategies to address the risks in high-risk occupations for Long COVID from a public health perspective. Clinically, careful evaluation of the return to work for patients experiencing high levels of workplace stress is important. Offering treatment plans that incorporate stress reduction strategies and psychological therapies before a return to work can mitigate the risk of Long COVID symptoms and support a healthier transition back into the workplace.

### 4.1. Limitations

There are several limitations to this survey. First, there is a possibility of selection bias because the study was conducted using an internet survey. Participants were workers who were able to register and respond to the internet survey, and thus any generalizations should be made with caution. Second, it is possible that workplace stressors may have changed over the 2-year period, leading to potential misclassification that could have resulted in an underestimation, even though the association remains valid. Third, confounding factors were not fully considered, including the variants of the infection,^40^ preexisting physical comorbidities similar to Long COVID symptoms, and the acute severity and duration following infection. Fourth, the validity of self-reported infection was uncertain. To verify the accuracy of self-reported PCR and positive antigen tests, the study included questions about experiences with hospitalization, facilities care, and home care. In Japan, individuals infected with COVID-19 were required to be hospitalized, treated at designated facilities, or undergo home quarantine. The agreement rate between these experiences and the reported test results was 98%, indicating strong validity. Fifth, fewer than half of the participants completed the follow-up survey. Although there were no significant differences between the follow-up group and those lost to follow-up, there were differences in several items. The profile of those lost to follow-up showed a higher smoking rate and higher job demands (Table S1), but it is unclear how these differences affected the results.

## 5. Conclusions

In this cohort study, we identified an association between workplace stressors prior to infection and the development of Long COVID in a large group of workers across Japan. These findings may contribute to elucidating the mechanism of the disease process of Long COVID. In addition, the results suggest that managing workplace stressors may contribute to preventing the development of Long COVID, and indicate the importance of workplace stressor management along with workplace infection control in the COVID pandemic.

## Supplementary Material

Web_Material_uiae062
